# Nardosinone Suppresses RANKL-Induced Osteoclastogenesis and Attenuates Lipopolysaccharide-Induced Alveolar Bone Resorption

**DOI:** 10.3389/fphar.2017.00626

**Published:** 2017-09-12

**Authors:** Chenguang Niu, Fei Xiao, Keyong Yuan, XuChen Hu, Wenzhen Lin, Rui Ma, Xiaoling Zhang, Zhengwei Huang

**Affiliations:** ^1^Shanghai Key Laboratory of Stomatology, Department of Endodontics, Ninth People’s Hospital, Shanghai Jiao Tong University School of Medicine Shanghai, China; ^2^Department of Orthopedic Surgery, Xin Hua Hospital Affiliated to Shanghai Jiao Tong University School of Medicine Shanghai, China

**Keywords:** nardosinone, RANKL, osteoclastogenesis, alveolar bone resorption, MAPKs

## Abstract

Periodontitis is a chronic inflammatory disease that damages the integrity of the tooth-supporting tissues, known as the periodontium, and comprising the gingiva, periodontal ligament and alveolar bone. In this study, the effects of nardosinone (Nd) on bone were tested in a model of lipopolysaccharide (LPS)-induced alveolar bone loss, and the associated mechanisms were elucidated. Nd effectively suppressed LPS-induced alveolar bone loss and reduced osteoclast (OC) numbers *in vivo.* Nd suppressed receptor activator of nuclear factor-κB ligand (RANKL)-mediated OC differentiation, bone resorption, and F-actin ring formation in a dose-dependent manner. Further investigation revealed that Nd suppressed osteoclastogenesis by suppressing the ERK and JNK signaling pathways, scavenging reactive oxygen species, and suppressing the activation of PLCγ2 that consequently affects the expression and/or activity of the OC-specific transcription factors, c-Fos and nuclear factor of activated T-cells cytoplasmic 1 (NFATc1). In addition, Nd significantly reduced the expression of OC-specific markers in mouse bone marrow-derived pre-OCs, including *c-Fos*, cathepsin K (*Ctsk*), *VATPase d2*, and *Nfatc1*. Collectively, these findings suggest that Nd has beneficial effects on bone, and the suppression of OC number implies that the effect is exerted directly on osteoclastogenesis.

## Introduction

Periodontitis is a chronic inflammatory disease that damages the integrity of the tooth-supporting tissues, known as the periodontium, and comprising the gingiva, periodontal ligament and alveolar bone (Hajishengallis, 2015). Oral bacteria in subgingival plaque have been identified as the primary etiological agent (Madianos et al., 2005), with *Porphyromonas gingivalis* lipopolysaccharide (LPS) (*P. gingivalis* LPS) identified as a key stimulus (Holt et al., 1988). Not only is periodontitis instigated by local dysbiotic microbial communities, but it is also the host inflammatory response to this microbial challenge that in the long run causes tissue damage, including pathologic activation of osteoclast cells (OCs) to resorb bone (Lamont and Hajishengallis, 2015). Normally, alveolar bone is constantly reconstructed by means of the balanced activities of OCs and osteoblasts (Hadjidakis and Androulakis, 2006). However, in periodontitis, OC activity is increased in the presence of pro-inflammatory cytokines produced by inflammatory cells and LPS produced by bacteria. This leads to alveolar bone loss, resulting in early tooth loss (Pihlstrom et al., 2005).

Osteoclast cells are best known as multinucleated giant cells derived from the monocyte/macrophage lineage. Their exclusive function is to resorb bone in response to macrophage colony-stimulating factor (M-CSF) and RANKL signaling generated from the bone microenvironment (Boyle et al., 2003; Asagiri and Takayanagi, 2007). The cytokine M-CSF is a prerequisite for providing proliferation and survival signals to OC precursor cells and increasing expression of receptor activator of nuclear factor kappa B (RANK), which is essential for OC differentiation (Boyle et al., 2003). Upon the binding of RANKL to RANK, tumor necrosis factor receptor-associated factor 6 (TRAF6) is invoked, resulting in a long series of downstream signaling cascades, including activation of the NF-κB signaling pathway and the mitogen-activated protein kinase (MAPK) signaling pathways. The signaling cascades conclude with the activation of c-Fos and nuclear factor of activated T cells cytoplasmic 1 (NFATc1), which are vital for osteoclastogenesis (Udagawa et al., 1999; Teitelbaum, 2000; Asagiri and Takayanagi, 2007). During RANKL-mediated osteoclastogenesis, it has been revealed that reactive oxygen species (ROS) play important roles in the differentiation, survival, activation, and bone resorptive activities of OCs (Garrett et al., 1990; Bhatt et al., 2002; Ha et al., 2004; Lee et al., 2005). In addition, excessive ROS generation has been associated with estrogen-deficient osteoporosis (Lean et al., 2003; Manolagas, 2010). The Ca^2+^-NFATc1 signaling pathway plays an important role in osteoclastogenesis, namely, the upregulation of intracellular Ca^2+^, which is dependent on the phosphorylation of phospholipase Cγ (PLCγ). PLCγ is essential for the activation of NFATc1 (Negishi-Koga and Takayanagi, 2009; Kim et al., 2014; Kim J.Y. et al., 2015). Intracellular Ca^2+^ and ROS have been revealed to upregulate and auto-amplify NFATc1, the master regulator of osteoclastogenesis, through the CaMKIV/CREB pathway (Hwang and Putney, 2011; Li P. et al., 2014). Accordingly, numerous biological compounds targeting modulation of the above signaling pathways involved in OC differentiation have been found to have the ability to ameliorate periodontal damage, especially alveolar bone loss (Kim Y.G. et al., 2015; Bhattarai et al., 2016). Therefore, screening active compounds which can promote the healing and regeneration of periodontal tissues or attenuate the injury of periodontitis is an effective strategy for the treatment of OC-related periodontal diseases.

Nardosinone (Nd), isolated from Nardostachys root, an important Chinese herbal medicine, has been reported to be an enhancer of nerve growth factor (Li et al., 1999). Several studies have proven that Nd possesses a wide range of pharmacological effects, including sedative, adaptogen-like, anti-depressive, anti-leukemic, anti-tumorous, and anti-trypanosomal activities (Otoguro et al., 2011; Li Z.H. et al., 2014; Ju et al., 2015; Kapoor et al., 2017). Interestingly, Nd was found to effectively suppress osteoclastogenesis in our previous screening work of single compounds extracted from Chinese herbs. However, the role of Nd on OC differentiation, as well as the underlying mechanisms through which osteoclastogenesis is regulated, have not been fully examined so far. In the present study, we confirmed that Nd can suppress the generation and differentiation of OCs from mouse bone marrow macrophages (BMMs) through JNK, ERK, PLCγ2, c-Fos, and NFATc1 signaling pathways in association with scavenging the RANKL-induced ROS. Furthermore, the defensive effect of Nd on *P. gingivalis* LPS-induced alveolar bone loss was evaluated in a mouse periodontitis model. These data add substance to the suggestion that Nd can help prevent inflammatory bone loss.

## Materials and Methods

### Reagents and Antibodies

**FIGURE 1:**
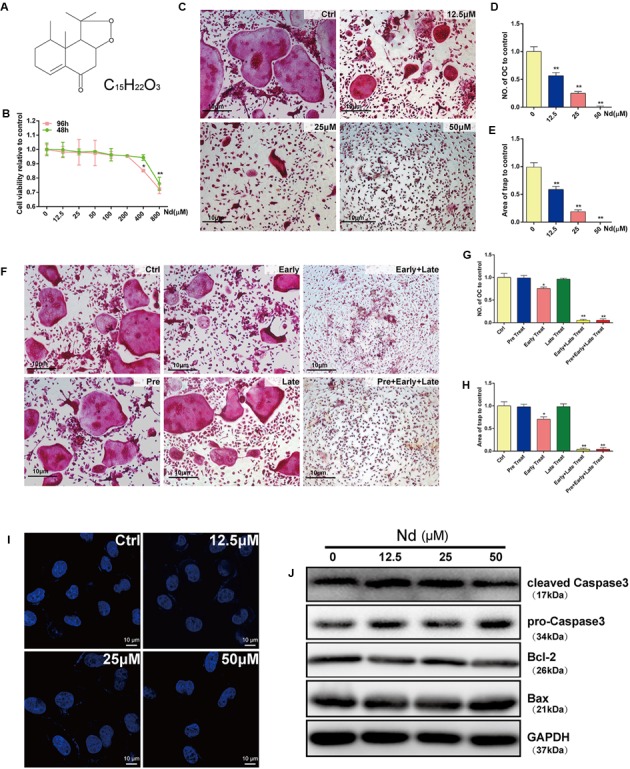
Evaluation of Nardosinone (Nd) toxicity and its effects on RANKL-induced osteoclastogenesis and apoptosis *in vitro*. **(A)** Chemical formula of Nd. **(B)** Cell viability of mouse bone marrow macrophages (BMMs) treated with varying doses of Nd (0, 12.5, 25, or 50 μM) for 48 or 96 h. **(C)** Typical images of BMMs stained for TRAP (red) after treatment with different concentration of Nd. **(D)** The number of TRAP^+ve^ multinucleated osteoclasts (≥3 nuclei) and the area **(E)** of TRAP compared to control were quantified. **(F)** The effect of time of addition of Nd on osteoclast formation. BMMs were stimulated with RANKL (100 ng/mL) alone or together with Nd (50 μM) at different stages of the 5-day osteoclast culture as described in Section “Materials and Methods.” **(G)** The number of TRAP^+ve^ multinucleated osteoclasts (≥3 nuclei) and the area **(H)** of TRAP compared to control were quantified. **(I)** The nuclear size and shape of BMMs at concentrations that affect OC formation and activity. Nd did not induce nuclear fragmentation. OCs cultured on glass coverslips were incubated with Nd at different doses (0, 12.5, 25, or 50 μM) for 48 h then fixed and stained with DAPI and examined by fluorescence microscopy. **(J)** Western blotting to check whether Nd induces apoptosis at concentrations that affect OC formation and activity. Values are expressed as mean ± SD; ^∗^*P* < 0.05, ^∗∗^*P* < 0.01 compared to control.

Nardosinone (**Figure 1A**), purchased from Must Biotechnology (Chengdu, China), was dissolved in dimethyl sulfoxide (DMSO). Alpha modification of Eagle’s minimum essential medium (α-MEM), fetal bovine serum (FBS), and penicillin/streptomycin were purchased from Gibco BRL (Gaithersburg, MD, United States). Recombinant murine M-CSF and RANKL were purchased from R&D Systems (Minneapolis, MN, United States). Tartrate-resistant acid phosphatase (TRAP) staining solution, Triton X-100, and 4′,6-diamidine-2′-phenylindole dihydrochloride (DAPI) were obtained from Sigma-Aldrich (St. Louis, MO, United States). FITC phalloidin was obtained from Yeasen biotech Co., Ltd (Chengdu, China). Primary antibodies targeting GAPDH, IκBα, phospho-Akt, Akt, phospho-ERK1/2, ERK1/2, phospho-JNK1/2, JNK1/2, phospho-p38, p38, phosphor-PLCγ2, PLCγ2, c-Fos and NFATc1 were purchased from Cell Signaling Technology (Danvers, MA, United States). Dichlorofluorescin diacetate (DCFDA) cellular ROS detection assay kits were obtained from Beyotime Institute of Biotechnology (Jiangsu, China). LPS from *P. gingivalis* was purchased from InvivoGen (San Diego, CA, United States).

### Cell Culture

Bone marrow macrophages were prepared as previously described (Xiao et al., 2015). Briefly, monocyte/macrophage precursors were prepared by flushing the marrow from the long bones of 6-week-old C57BL/J6 mice and differentiated into BMMs in α-MEM containing 10% FBS, 100 U/mL penicillin/streptomycin (complete α-MEM) and 30 ng/mL M-CSF. The OC-like RAW264.7 cell line (TIB-71, ATCC, Manassas, VA, United States) was cultured in complete α-MEM. All cell cultures were maintained in a humidified environment of 95% air/5% CO_2_ at 37°C.

### Cell Viability Assay

The anti-proliferative effect of Nd on BMMs was measured with a CCK-8 kit (Dojindo Molecular Technology, Kumamoto, Japan) according to the manufacturer’s instructions. In brief, BMMs plated into 96-well plates at a density of 1 × 10^4^ cells/well were treated for 48 or 96 h with serial dilutions of Nd (0–800 μM). Next, 10 μL CCK-8 mixed with 90 μL complete α-MEM was added to each well. After incubation for 2 h, the optical density (OD) was measured with an ELX800 absorbance microplate reader (Bio-Tek Instruments, Inc., Winooski, VT, United States) at a wavelength of 450 nm (650 nm reference).

### Analysis of DAPI Staining

Bone marrow macrophages were treated with Nd (0, 12.5, 25, or 50 μM) for 48 h. The cells were washed three times with PBS, then treated for 15 min with Triton X-100 to disrupt the cell membrane integrity. Nuclei were stained with 0.1 μg/mL DAPI in PBS at 37°C for 10 min in the dark. The cell nuclei were observed and photographed using an LSM5 confocal microscope (Carl Zeiss, Oberkochen, Germany).

### *In Vitro* Osteoclastogenesis Assay

Bone marrow macrophages were seeded into 96-well plates at a density of 8 × 10^3^ cells/well, in triplicate, in complete α-MEM containing M-CSF (30 ng/mL), and allowed to adhere overnight. Next day BMMs were treated with RANKL (50 ng/mL) and various concentrations of Nd (0, 12.5, 25, or 50 μM) over a 5 day period. Conversely, to investigate Nd addition-dependent effects on osteoclastogenesis at a particular stage, Nd (50 μM) was added to BMMs cultured with M-CSF and RANKL at different stages of the 5 day culture period as follows: pre-treatment (12 h before RANKL); early treatment (day 1); late treatment (day 3), early + late treatment (days 1 and 3); and pre + early + late treatment (12 h before RANKL, then days 1 and 3). After 5 days, cells were fixed with 4% paraformaldehyde and stained for TRAP activity. TRAP^+ve^ cells that contained three or more nuclei were counted as mature OCs, and their cell spread areas were measured.

### Quantitative Polymerase Chain Reaction (PCR) Analysis

For real-time polymerase chain reaction (PCR), 2 × 10^5^ BMMs were plated into each well of a 12-well plate and cultured in complete α-MEM containing M-CSF (30 ng/mL), and RANKL (50 ng/mL). Cells were then treated with or without Nd at a range of concentrations (0, 12.5, 25, or 50 μM). Total RNA was extracted from cultured cells using an RNeasy Mini kit (Qiagen, Valencia, CA, United States) according to the manufacturer’s instructions, and cDNA was synthesized from 1 μg of total RNA using reverse transcriptase (TaKaRa Biotechnology, Otsu, Japan). Real-time PCR was performed using a SYBR Premix Ex Taq kit (TaKaRa Biotechnology) and an ABI 7500 Sequencing Detection System (Applied Biosystems, Foster City, CA, United States). PCR was performed under the following conditions: 40 cycles each involving 5 s of denaturation at 95°C and 34 s of amplification at 60°C. All PCRs were performed in triplicate and levels were normalized to those of the gene *Gapdh*. The following primer sets were used as previously described: mouse *Gapdh*: forward, 5′-ACC CAG AAG ACT GTG GAT GG-3′ and reverse, 5′-CAC ATT GGG GGT AGG AAC AC-3′; mouse *Nfatc1*: forward, 5′-CCG TTG CTT CCA GAA AAT AAC A-3′ and reverse, 5′-TGT GGG ATG TGA ACT CGG AA-3′; mouse *cathepsin K*: forward, 5′-CTT CCA ATA CGT GCA GCA GA-3′ and reverse, 5′-TCT TCA GGG CTT TCT CGT TC-3′; mouse *c-Fos*: forward, 5′-CCA GTC AAG AGC ATC AGC AA-3′, reverse, 5′-AAG TAG TGC AGC CCG GAG TA-3′; and mouse *V-ATPase d2*: forward, 5′-AAG CCT TTG TTT GAC GCT GT-3′ reverse, 5′-TTC GAT GCC TCT GTG AGA TG-3′.

### Western Blot Analysis

RAW264.7 cells were seeded into 6-well plates at a density of 6 × 10^5^ cells/well. When the cells were confluent, they were pretreated with or without Nd for 4 h. Cells were then stimulated with 50 ng/mL RANKL for 0, 5, 10, 20, 30, and 60 min. Cellular proteins were extracted from cultured BMMs or RAW264.7 cells using RIPA lysis buffer (Thermo Fisher Scientific, Waltham, MA, United States) supplemented with 10 mg/mL phenylmethylsulfonyl fluoride (PMSF), and the protein concentration was determined using a BCA protein assay (Thermo Fisher Scientific). Lysate proteins (25 μg) were separated by 10% SDS-PAGE and transferred to polyvinylidene difluoride membranes. Membranes were then blocked with 5% skimmed milk in TBS-Tween (TBS: 0.05 M Tris, 0.15 M NaCl pH 7.5; with 0.2% Tween-20) for 1 h, and incubated with primary antibodies diluted in 1% (w/v) skimmed milk powder in TBS-Tween overnight at 4°C. Experiments were repeated independently at least three times.

### Bone Resorption Pit Assay

Equivalent amounts of BMM-derived pre-OCs (after 4 days of RANKL stimulation) were seeded onto 100-mm bovine bone disks, treated with Nd (0, 12.5, 25, or 50 μM) for 48 h and then fixed and stained for TRAP activity. Resorption pits were visualized under a scanning electron microscope (FEI Quanta 250), and the bone resorption area was quantified using Image J software (National Institutes of Health, Bethesda, MD, United States).

### Immunofluorescence Confocal Microscopy

Osteoclast cells differentiated from BMMs were cultured on glass coverslips and fixed for 15 min at RT with 4% paraformaldehyde, permeabilized for 5 min with 0.1% Triton X-100 in PBS, and non-specific antibody binding was blocked by incubating for 30 min in 5% skimmed milk in PBS. F-actin was stained with FITC-conjugated phalloidin and nuclei were stained with DAPI. Actin ring distribution was visualized using an LSM5 confocal microscope (Carl Zeiss, Oberkochen, Germany).

### Intracellular ROS Detection

The DCFDA cellular ROS detection assay kit was used to detect intracellular ROS levels. BMMs (8 × 10^3^ cells/well in 96-well plates) were treated with RANKL (50 ng/mL), M-CSF (30 ng/mL), and Nd (25 or 50 μM) for 72 h. Intracellular ROS levels were determined using 2′,7′-dichlorofluorescein diacetate (DCFH), which oxidizes into fluorescent DCF in the presence of ROS. Cells were washed in PBS and incubated in the dark for 60 min with 10 μM DCFH-DA. Images were obtained using a fluorescence microscope (Carl Zeiss, Oberkochen, Germany).

### Alveolar Bone Resorption Experiments

Animal studies were conducted in accordance with the principles and procedures approved by the Animal Care Committee of Shanghai Jiao Tong University, China. Twenty specific-pathogen-free 8-week-old male C57BL/6J mice were randomly divided into four groups: phosphate-buffered saline (PBS; control), LPS (1 mg/kg body weight; Sigma-Aldrich), LPS + Nd (5 mg/kg body weight, low dose group), and LPS + Nd (15 mg/kg body weight, high dose group). Nd was given by intraperitoneal injection on days 1, 4, and 7. On days 4 and 7, mice were sedated via light anesthesia and injected with 1 mg/kg of *P. gingivalis* LPS at the gingiva of the second molar in the lower left and right jaw (Kim Y.G. et al., 2015). Mice were sacrificed on day 10 and their jaws were collected for further analysis. Left jaws were dissected for micro-CT analysis and right jaws for bone histology and histomorphometry.

### MicroCT Analysis of Jaws

Left jaws were fixed in 4% paraformaldehyde for 1 day at 4°C and stored in 70% ethanol at 4°C. They were then scanned using high-resolution micro-computed tomography (μCT) (Scanco microCT100, Brüttisellen, Switzerland). Each jaw was then imaged with the following instrument settings: 70 kV, 200 μA, 0.5 mm Al filter, 300 ms exposure, pixel size 5 μm. After scanning, the data were reconstructed using Scanco μ100 Evaluation software V6.5-3 with a constant threshold value. An image of a precise ruler was captured at the same magnification and used for calibration area measurements, which were performed with Olympus Microsuite 3.2 imaging software. A volume of interest was generated covering the first to third molars and the relevant alveolar bone. The volume of alveolar bone included in the region of interest (bone volume/total volume) (ROI [BV/TV]) was measured for each sample, and comparisons were made among different groups. The polygonal area enclosed by the cementoenamel junction (CEJ), the lateral margins of the exposed tooth root, and the alveolar bone crest (ABC) were also measured, and results are expressed in millimeters squared (mm^2^).

### Histological and Histomorphometric Analysis

Right jaws were fixed in 4% paraformaldehyde for 1 day at 4°C, subsequently decalcified in 10% EDTA for approximately 1 month, and then embedded in paraffin. Histological sections (7 μm thick) were prepared for hematoxylin and eosin as well as TRAP staining. The specimens were then examined and photographed using a high quality microscope. The number of TRAP^+ve^ multi-nucleated OCs (N.Oc/BS, 1/mm) and the percentage of OC surface per bone surface (OcS/BS, %) were assessed for each sample.

### Statistical Analysis

Data are shown as mean ± standard deviation (SD) from at least three independent experiments. Student’s *t*-test was used to determine statistical significance between the results of test and control groups, with ^∗^*P* < 0.05 and ^∗∗^*P* < 0.01 considered statistically significant.

## Results

### Nd Suppressed RANKL-Induced Osteoclastogenesis without Cytotoxic Effects

The CCK-8 assay was used to examine the potential cytotoxicity of Nd on BMMs. Treatment with Nd for 48 or 96 h at concentrations less than 400 μM did not induce cytotoxicity (**Figure 1B**). BMMs were exposed to M-CSF and RANKL in the absence or presence of different concentrations of Nd for 5 days. The formation of TRAP^+ve^ OCs was suppressed by Nd (**Figure 1C**) and the number of OCs and the area of TRAP-positive staining were reduced to approximately 9% of control levels by treatment with 50 μM Nd (**Figures 1D,E**). To determine the stage at which Nd suppressed osteoclastogenesis, BMMs were treated with 50 μM Nd at several time points. Neither treatment for 12 h prior to RANKL stimulation (pre-treatment) nor on the third day of RANKL stimulation (late treatment) altered the number or size of TRAP^+ve^ OCs induced after 5 days of culture, whereas treatment on the first day of RANKL stimulation (early treatment) led to an almost 35% reduction in OC number and area of TRAP (**Figures 1F–H**). Continuous exposure to early and late treatment or pre-, early and late treatment potently suppressed OC formation (**Figures 1F–H**). To exclude the possibility that Nd induced BMM apoptosis during the course of differentiation, BMMs were stained with DAPI following Nd treatment (**Figure 1I**), and were found to exhibit normal intact nuclei, confirming that the suppressory effects of Nd on osteoclastogenesis were not attributable to the induction of BMM apoptosis. Furthermore, western blotting was carried out to examine whether the apoptotic cell death pathways were activated after Nd treatment. There was no alteration in the levels of the anti-apoptotic protein Bcl-2, nor any activation of the Bax and caspase-3 apoptotic pathways following treatment with up to 50 μM of Nd (**Figure 1J**).

### Nd Decreased Osteoclastic Bone Resorption and F-Actin Ring Formation

**FIGURE 2:**
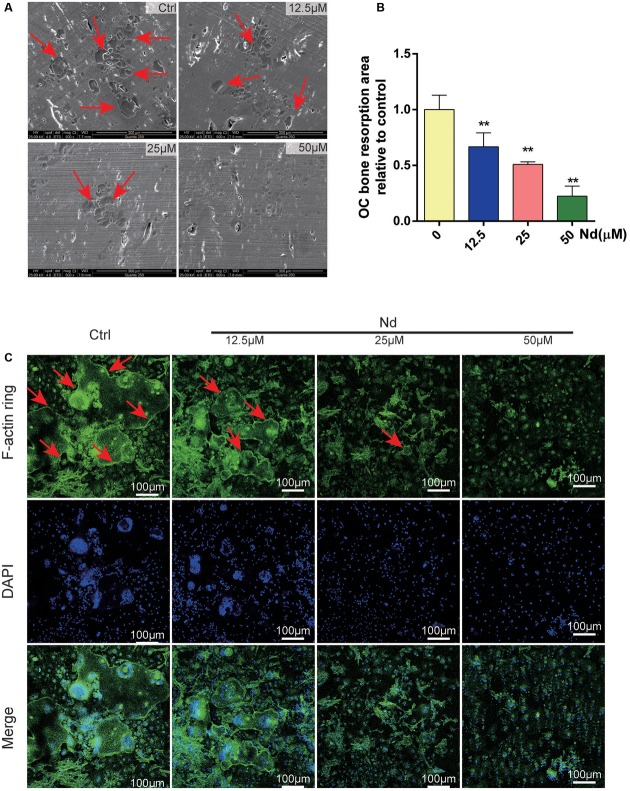
Nd attenuated osteoclastic bone resorption and F-actin ring formation *in vitro*. **(A)** Representative SEM images of bone resorption pits (red arrows) are shown following treatment with various doses of Nd (0, 12.5, 25, or 50 μM). **(B)** The total areas of resorption pits were quantified. **(C)** BMMs were incubated with M-CSF (30 ng/mL) and RANKL (100 ng/mL) and then treated with varying doses of Nd (0, 12.5, 25, or 50 μM). Confocal microscopy revealed clearly the formation and morphology of the F-actin (red arrows). Data are expressed as mean ± SD; ^∗∗^*P* < 0.01 compared to control.

Next, the effects of Nd on OC bone resorption were assessed. SEM results verified the bone resorptive ability of OCs differentiated from BMMs on the bone surface (**Figure 2A**) Meanwhile, most of the bone resorption activity was suppressed and was almost completely blocked at the higher concentration of Nd (≥50 μM) (**Figures 2A,B**). The establishment of F-actin-rich podosomes-polarized cytoskeletal structures known as F-actin rings-indicates the maturity of OCs and is also essential for OC bone resorption (Wu et al., 2015; Zhu et al., 2016). The results showed that osteoclastic bone resorption is suppressed by Nd; further experimentation to verify whether Nd suppresses F-actin ring formation was carried out. Confocal microscopy revealed clearly the formation and morphology of the F-actin ring of mature OCs stained with phalloidin in the control group (**Figure 2C**). However, in the group treated with Nd the size and morphology of the F-actin ring were reduced, with dose-dependent reductions. Together, these findings clearly indicate that the application of Nd reduced osteoclastic bone resorption and formation of OCs *in vitro*.

### Nd Eliminated ROS Production and Suppressed Activation of PLCγ2

**FIGURE 3:**
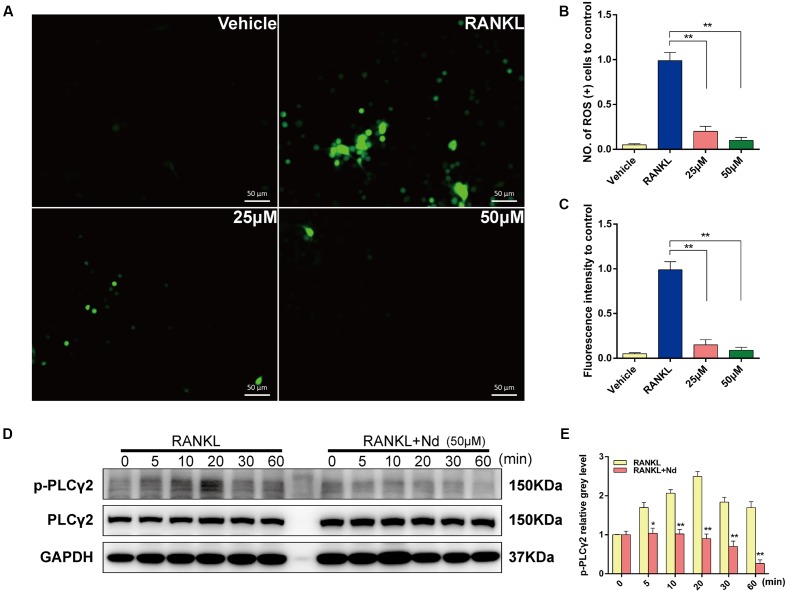
Nd scavenged ROS production and suppressed the activation of PLCγ2. **(A)** Representative images of ROS(+) BMMs during RANKL-induced osteoclastogenesis treated with varying concentrations of Nd (25, 50 μM); The ROS-positive cell numbers **(B)** and green fluorescence intensity **(C)** in each well (96-well plate) were quantified. **(D)** Western blot analysis of p-PLCγ2 protein expression in BMMs treated with or without Nd for different time periods. **(E)** The band intensities corresponding to p-PLCγ2 were quantified and normalized relative to that of GAPDH and converted to fold change of control. Data are expressed as mean ± SD. ^∗^*P* < 0.05, ^∗∗^*P* < 0.01 compared to the RANKL-induced group.

To elucidate the inhibitory effects of Nd on OC formation, intracellular ROS was semi-quantitatively measured using DCFDA. As shown in **Figure 3A**, intracellular ROS was at a low level before RANKL stimulation but increased sharply to a high level when BMMs were treated with RANKL. This effect could be markedly attenuated by Nd. Both the number of ROS (+) cells and the level of ROS were decreased by Nd (**Figures 3B,C**). Ca^2+^ signaling is indispensable for osteoclastogenesis and its trigger is the phosphorylation of PLCγ2. Thus, we investigated the inhibitory effects of Nd on the phosphorylation of PLCγ2. As expected, the phosphorylation of PLCγ2 was clearly blocked compared with the control group (**Figures 3D,E**).

### Nd Inhibited RANKL-Induced ERK and JNK1/2 Activation

**FIGURE 4:**
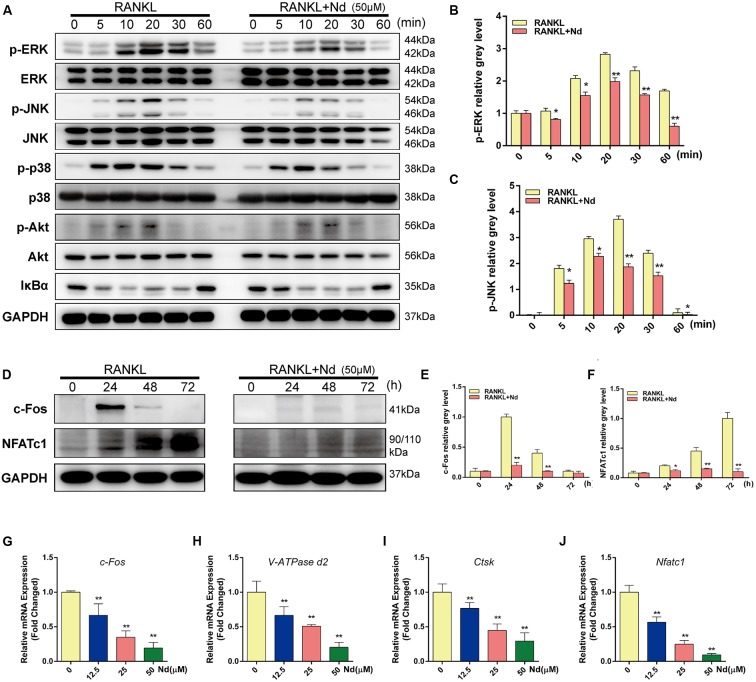
Nd specifically decreased RANKL-induced ERK and JNK cascades and suppressed RANKL-induced activation of c-Fos and NFATc1. **(A,D)** BMMs were treated with or without 50 μM Nd and then treated with 100 ng/mL RANKL for the indicated periods. Cell lysates were analyzed using western blotting. The band intensities corresponding to p-ERK **(B)**, p-JNK **(C)**, c-Fos **(E)**, and NFATc1 **(F)** were quantified. Nd inhibited the RANKL-induced expression of osteoclast-specific genes and real-time PCR was used to determine the expression of osteoclast marker genes *c-Fos* **(G)**, *V-ATPase d2* **(H)**, *Ctsk* **(I)**, and *Nfatc1* **(J)**. Data are expressed as mean ± SD. ^∗^*P* < 0.05, ^∗∗^*P* < 0.01 compared to the RANKL-induced group.

To detect the underlying mechanisms by which Nd mediated OC formation, the bearing of RANKL-induced signaling pathways on osteoclastogenesis were examined. The phosphorylation of MAPK family members (EKR, JNK, and p38) and Akt occurred within 10 min of RANKL stimulation, and continued for 30 min in the control group (**Figures 4A–C**). In the meantime, by comparison, Nd attenuated the phosphorylation of ERK and JNK, without affecting that of p38 (**Figure 4A**). The results also showed that Nd did not reduce phosphorylation of Akt or of IκBα, an inhibitory subunit of NFκB, indicating that it does not influence the Akt and NFκB signaling pathway (**Figure 4A**). Overall, these data suggest that the suppressive influence of Nd on osteoclastogenesis could be put down to the attenuation of RANKL-induced JNK and ERK signaling cascades.

### Nd Suppressed the Activation of c-Fos and NFATc1

It has been shown that activation of NFATc1, the master regulator of osteoclastogenesis which relies on c-Fos, results from phosphorylation of the MAPK signaling pathway (Nakashima et al., 2012). BMMs were stimulated with RANKL in the absence or presence of Nd for 0, 24, 48, or 72 h. As shown in **Figures 4D–F**, c-Fos protein expression increased after 24, 48, and 72 h, and NFATc1, the downstream transcriptional targets of c-Fos, showed steep increases at 48 and 72 h. By comparison, the situation was potently suppressed by Nd at various time-points. (**Figures 4G–J** On the other hand, Nd also suppressed the expression of OC marker genes including *c-Fos*, *VATPase d2*, *ctsk*, and *Nfatc1*), shown by quantitative PCR.

### Nd Attenuated LPS-Stimulated Bone Loss

**FIGURE 5:**
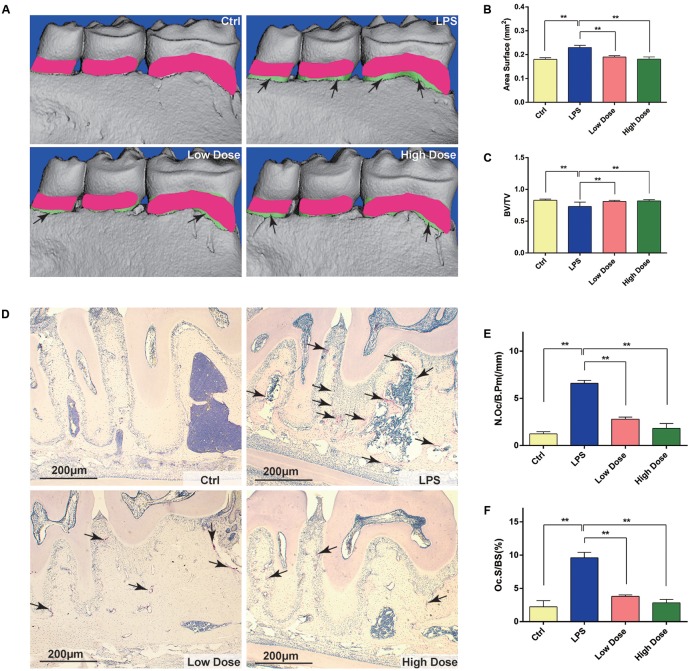
Nd protects against LPS-induced alveolar bone loss by inhibiting osteoclast activity. **(A)** Lingual view of representative 3D constructions of the hemi-mandible from the control group, LPS-stimulated group, low dose group, and high dose group. The green area represents the difference compared with the control group. **(B)** Measurement of bone levels was performed by comparing the area of exposed roots of the three molars on the 3D constructions. **(C)** Quantitative analyses of bone volume/total volume. **(D)** Representative images of decalcified alveolar bone stained with TRAP (black arrows) from the control group, LPS-stimulated group, low dose group, and high dose group. **(E)** Quantitative analyses of osteoclast surface/bone surface (Oc.S/BS). **(F)** Quantitative analyses of osteoclast number/bone surface (N.Oc/BS). *n* = 5. Data are expressed as mean ± SD; ^∗∗^*P* < 0.01 relative to LPS-stimulated group.

To evaluate the role of Nd in bone resorption *in vivo*, we created the LPS-induced mouse alveolar-bone-loss model. No fatalities were recorded after LPS and Nd administration, and all the animals maintained normal activity throughout the experiment. Micro-CT, morphometric and histomorphometric analyses were performed. Micro-CT confirmed that LPS-injected mice exhibited significantly greater areas of exposed roots than the control and Nd groups (**Figures 5A,B**). No significant differences in areas of exposed roots were observed among control, low dose, and high dose groups (**Figures 5A,B**). A volumetric quantitative analysis of alveolar bone loss with micro-CT verified the results obtained by macroscopic analysis. BV/TV in the LPS group was also significantly lower than those of the control or Nd groups (**Figure 5C**). The LPS-mediated decrease in BV/TV values was restored up to a level similar to the control group by treatment with Nd at 5 or 15 mg/kg (**Figure 5C**).

To further explore whether OCs were involved in the inhibitory effects of Nd on LPS-induced alveolar bone loss, TRAP staining was performed to calculate the number of OCs in tissue sections. TRAP-positive multinucleated cells lining the alveolar bone surface were visually enumerated (black arrows in **Figure 5D**) in a specific AOI with standardized dimensions and surfaces. The LPS group showed the highest number of OCs compared with the control group. However, the administration of 5 mg/kg of Nd significantly reduced OC number. Furthermore, the administration of 15 mg/kg of Nd almost completely prevented an LPS-induced increase in OC number and bone erosion (**Figure 5D**). Histomorphometric analysis of Oc.S/BS and OC number confirmed that Nd treatment attenuated LPS-induced alveolar bone loss and reduced OC numbers (**Figures 5E,F**). Collectively, these results indicated that Nd effectively prevented LPS-induced alveolar bone loss *in vivo*. Tissue toxicities of Nd on the liver and kidney were confirmed by histological analyses (**Supplementary Figure S1**). Compared to control animals, administration of Nd at 5 or 15 mg/kg body weight/day for 10 days did not induce visible hepatotoxicity or nephrotoxicity.

## Discussion

Bone resorption is a major pathological factor in periodontitis and it is now clear that deregulation of immune and inflammatory responses is crucial in initiating the bone destruction associated with these conditions (Jimi et al., 2004). In the present study, our data show that Nd efficiently suppresses *P. gingivalis* LPS-induced alveolar bone loss, demonstrated by direct microCT analyses and histology *in vivo*, and suppressed osteoclastogenesis *in vitro*. To our knowledge, this is the first study to explore the effects of Nd on bone metabolism. At the molecular level, Nd profoundly suppressed multiple pathways downstream of RANK, including ROS, PLCγ2, MAPKs, c-Fos, and NFATc1.

In the present study, the inhibitory effect of Nd on RANKL-induced OC formation from BMMs with RANKL and M-CSF was first explored *in vitro* and *in vivo*. It is well-known that RANKL, together with M-CSF, is a prerequisite for osteoclastogenesis (Boyle et al., 2003) and OCs are differentiated from hematopoietic cells through a multi-step process, including proliferation, expression of TRAP, and fusion of cells, and are TRAP^+ve^ multinucleated cells with bone resorbing activity (Takahashi et al., 1999). Then, based on these theories, the results in **Figures 1C–H** suggest that the effects of Nd do not last long, and treatment at the early stages and during the course of RANKL-induced differentiation could produce a more effective inhibition of OC formation. The dose-dependent effect is transient since the removal of Nd in the early stage of osteoclastogenesis largely restored OC formation. A well-polarized F-actin ring is the most distinct characteristic of mature OCs and it is also essential for osteoclastic bone resorption (Wilson et al., 2009). The effect of Nd on F-actin ring formation and bone resorption ability are verified in our study.

Stimulation with RANKL has been shown to transiently increase the intracellular levels of ROS, which regulates RANK signaling pathways including Akt, MAPKs, and NFκB, thereby promoting osteoclastogenesis, while osteoclastogenesis is blocked entirely when ROS production is prevented (Lee et al., 2005). Our results described here show that Nd is more effective at inhibiting the generation and accumulation of ROS, indicating that Nd may also suppress osteoclastogenesis through antioxidation. ROS acts as a second messenger in signal transduction and gene regulation in a variety of cell types, and under the influence of several biological factors such as cytokine, growth factor, or hormone treatments (Lander, 1997; Hensley et al., 2000; Hadjidakis and Androulakis, 2006). Cells are capable of defending themselves against ROS damage with enzymes such as catalases, superoxide dismutases, glutathione peroxidases, glutathione reductase, and peroxiredoxins under normal circumstances (Finkel, 2003; Moon et al., 2006; Wauquier et al., 2009). The induction of oxidative stress is a potential mechanism by which periodontitis manifests its systemic effects (D’Aiuto et al., 2010). Previous studies point to the association of ROS with the pathogenesis of periodontitis (Waddington et al., 2000). Cellular ROS accumulation occurs during OC formation and, if it is prolonged and persistent, brings about the destruction of periodontal tissue and oxidative damage (Bhatt et al., 2002; Bhattarai et al., 2016). In addition, Altindag *et al.* reported that an accumulation of ROS and a lessened anti-oxidative status have been observed in osteoporosis patients (Altindag et al., 2008).

RANKL also triggers the phosphorylation of PLCγ2, which subsequently leads to Ca^2+^ mobilization in the process of OC formation (Negishi-Koga and Takayanagi, 2009). Ca^2+^ signaling is crucial for osteoclastogenesis. The transient initial release of Ca^2+^ from intracellular stores and the influx through specialized Ca^2+^ channels controls the dephosphorylation of NFATc1 protein, and leads to its nuclear localization, which is followed by the activation of OC-specific genes (Yeon et al., 2015). PLCγ2 phosphorylation is directly and closely coupled with Ca^2+^ oscillations and the Ca^2+^-dependent translocation of NFATc1 induced by RANKL (Takayanagi, 2007). In addition, it has been suggested that it is the phosphorylation of PLCγ2 rather than PLCγ1 that is required for RANKL-mediated Ca^2+^ signaling in OC differentiation (Mao et al., 2006). In light of the above, we speculated that the inhibitory effect of Nd on the phosphorylation of PLCγ2 is likely to suppress the RANKL-Ca^2+^-oscillation-NFATc1 activation signaling axis during OC differentiation. Further study is required to investigate whether Nd treatment reduces the amplitude and frequency of Ca^2+^ oscillations in the OC differentiation process.

One of the most important downstream pathways mediating the effects of Nd on osteoclastogenesis could be components of MAPK signaling, namely ERK and JNK. RANKL invokes the rapid phosphorylation and activation of MAPKs and AKT, which consequently stimulates the activation of transcription factors such as c-Fos and NFATc1 to regulate the expression of genes required for OC differentiation (Stevenson et al., 2011; Yeon et al., 2014). Increasing evidence shows that the RANKL-induced ERK signaling pathway plays an important role in osteoclastogenesis (Tsai et al., 2008; Tang et al., 2009). When ERK signaling is still active, c-Fos is phosphorylated by sustained ERK (Okawa et al., 2009). It is well-known that c-Fos is a member of the AP-1 transcription factor family and is required for the differentiation of OC precursors into bone-resorbing OCs. c-Fos-deficient mice suffer from osteopetrosis because of a block of OC differentiation. At the same time, by means of ectopic c-Fos expression, impaired osteoclastogenesis in BMMs is completely rescued (Grigoriadis et al., 1994). c-Fos regulates expression of NFATc1, which is critical for the differentiation of OCs. NFATc1-deficient mice have defects of osteoclastogenesis and also show symptoms of osteopetrosis (Winslow et al., 2006). Similarly, activated JNK phosphorylates downstream factors, including c-Fos, which is required for NFATc1 induction (Jimi et al., 1999; Takayanagi et al., 2000). Our data (**Figure 4**) suggest that Nd may act on ERK and JNK signaling pathways along the c-Fos/NFATc1 axis to interfere with osteoclastogenesis.

Erosion of alveolar bone is one of the grave consequences of periodontitis which is an inflammatory disease. For the moment, antiresorptive agent available for periodontitis is in urgent need, since bisphosphonates and anti-RANKL antibodies have the risk of increasing osteonecrosis of the jaw. In an LPS-induced mouse periodontitis model, the administration of Nd meaningfully reduced the number of osteoclasts as well as alveolar bone erosion (**Figure 5**) and this suggests Nd suppresses osteoclastogenesis and could play an effective role in depressing bone resorption *in vivo.* It is important to note the limitations within the present study that represent the future direction of our research. Firstly, whether Nd correlates negatively with these inflammatory cytokines should be explored in future studies, since IL-1, IL-6, IL-8, and TNF-α promote osteoclastogenesis and bone resorption through multiple mechanisms, such as increasing the production of M-CSF and RANKL (Koerner et al., 2008; Xu et al., 2011). Secondly, further study should focus on identifying the target binding molecules of Nd, and the mechanism via which Nd suppresses the fusion of pre-OCs and the pit formation of mature OCs might be elucidated.

Collectively, our data demonstrate that Nd can suppress osteoclastogenesis and periodontal bone loss. This effect is mediated by scavenging RANKL-induced ROS activity and the suppression of RANKL-stimulated activation of JNK, ERK, PLCγ2, c-Fos, and NFATc1 signaling pathways during OC formation and bone resorption. Taken together, our data suggest that Nd may represent a potential agent for the treatment of periodontitis or other OC-related osteolytic diseases.

## Author Contributions

All authors listed have made a substantial, direct and intellectual contribution to the work, and approved it for publication.

## Conflict of Interest Statement

The authors declare that the research was conducted in the absence of any commercial or financial relationships that could be construed as a potential conflict of interest.

## Supplementary Material

FIGURE S1 Nd toxicity on mouse liver and kidney.
