# Supplementary figures and images for: Nardosinone Suppresses RANKL-Induced Osteoclastogenesis and Attenuates Lipopolysaccharide-Induced Alveolar Bone Resorption
Source: Front Pharmacol. 2017 Sep 12;8:626. doi: 10.3389/fphar.2017.00626 (PMC5601052; doi:10.3389/fphar.2017.00626)

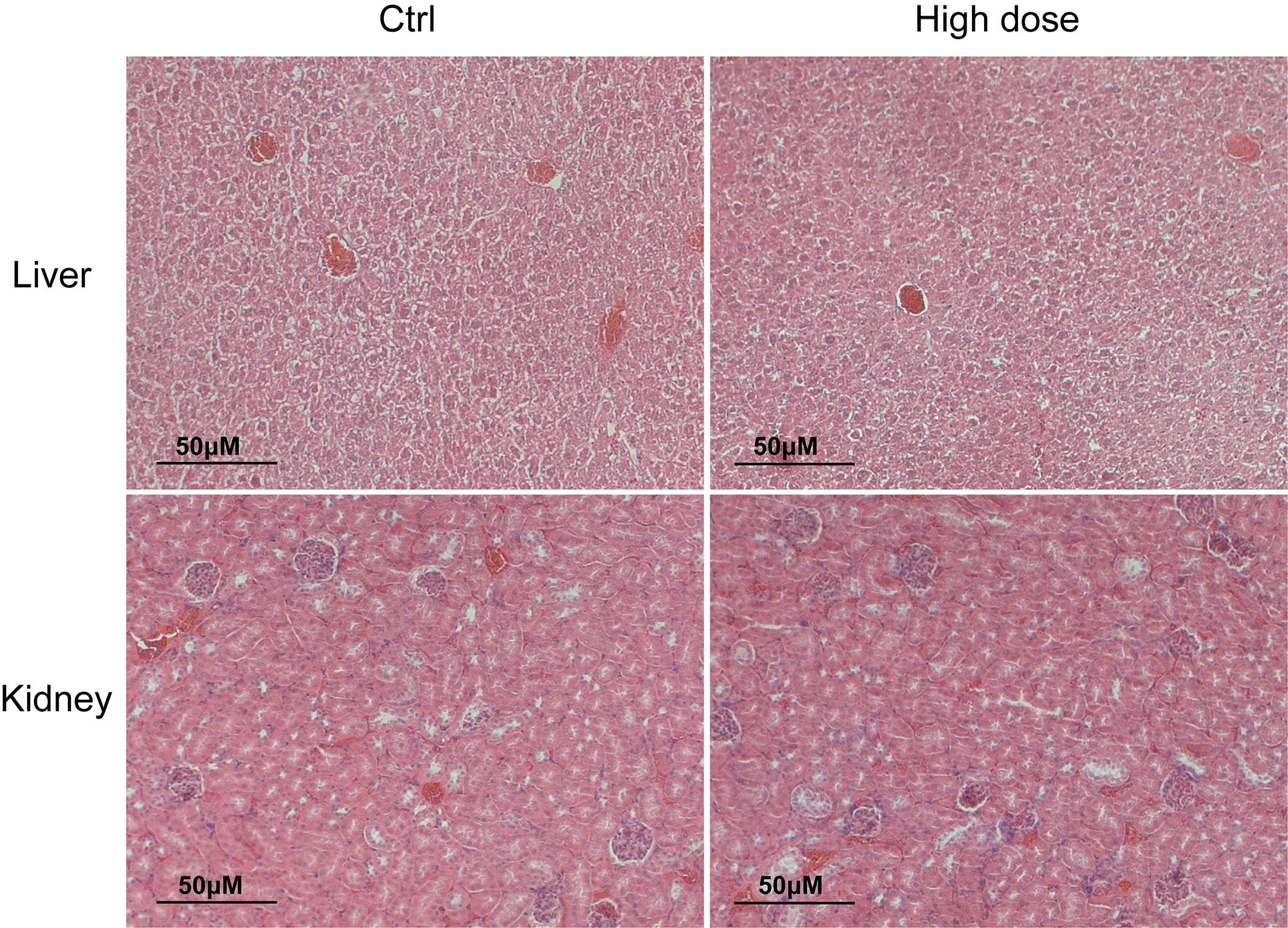

Supplement: FIGURE S1 — Nd toxicity on mouse liver and kidney. [file Image_1.JPEG]
